# The outer membrane autotransporters Fap2 and CmpA facilitate specific coaggregation between *Fusobacterium nucleatum* and *Aggregatibacter actinomycetemcomitans* serotypes b and d

**DOI:** 10.1128/aem.01132-25

**Published:** 2025-10-20

**Authors:** Yumika Tanaka, Yuichi Oogai, Airi Matsumoto, Kazuyuki Noguchi, Masanobu Nakata

**Affiliations:** 1 Department of Oral Microbiology, Kagoshima University Graduate School of Medical and Dental Sciences 208512 , Kagoshima, Japan; 2 Department of Periodontology, Kagoshima University Graduate School of Medical and Dental Sciences 208512 , Kagoshima, Japan; Washington University in St. Louis, St. Louis, Missouri, USA

**Keywords:** *Fusobacterium nucleatum*, *Aggregatibacter actinomycetemcomitans*, coaggregation

## Abstract

Periodontal disease is a polymicrobial infection in which *Fusobacterium nucleatum* and *Aggregatibacter actinomycetemcomitans* have been identified as key pathogens associated with disease progression. *F. nucleatum* is capable of coaggregating with several oral bacterial species, including *A. actinomycetemcomitans*, and these coaggregations play crucial roles in dental plaque formation. In this study, we investigated the coaggregation properties of both bacteria and identified the molecules that mediate their interaction. Among *A. actinomycetemcomitans* serotypes a, b, c, d, and g, *F. nucleatum* strains coaggregated specifically with serotypes b and d. The coaggregation between *F. nucleatum* and *A. actinomycetemcomitans* serotype b was significantly suppressed by the addition of lipopolysaccharide (LPS) extracted from *A. actinomycetemcomitans* serotype b. Among the sugars comprising the O-polysaccharide (O-PS) of serotype b LPS, the addition of *N*-acetyl-_D_-galactosamine markedly inhibited their coaggregation. Similarly, the coaggregation between *F. nucleatum* and *A. actinomycetemcomitans* serotype d was strongly inhibited by the addition of either the LPS extracted from *A. actinomycetemcomitans* serotype d or the _L_-rhamnose present in serotype d O-PS. Furthermore, analysis of outer membrane autotransporter proteins as potential coaggregation factors of *F. nucleatum* revealed that mutants lacking functional *fap2* or *cmpA* exhibited significantly reduced coaggregation with *A. actinomycetemcomitans* serotype b or d, respectively. Additionally, we found that these coaggregations significantly promoted biofilm formation during coculture. These findings suggest that the coaggregation between *F. nucleatum* and *A. actinomycetemcomitans* serotypes b and d is mediated through interactions between autotransporter proteins and LPSs, thereby contributing to the development of mature biofilms.

**IMPORTANCE:**

*Fusobacterium nucleatum* is a periodontal pathogen that acts as a bridging organism by coaggregating with various oral microbes, including other periodontal pathogens. Recently, this bacterium has been reported to be associated with systemic diseases, including cardiovascular diseases, adverse pregnancy outcomes, and cancer. *F. nucleatum* possesses numerous adhesins, including autotransporter proteins that are considered to mediate colonization in human tissues. Our study demonstrated that *F. nucleatum* utilizes two autotransporter proteins (Fap2 and CmpA) to coaggregate with another periodontal pathogen, *Aggregatibacter actinomycetemcomitans*, through specific interactions with bacterial surface polysaccharides. In addition to the roles of these autotransporters in periodontitis through bacterial coaggregation with other oral bacterial species, our findings potentially provide new insights into the mechanism underlying systemic diseases caused by *F. nucleatum* binding to host cell carbohydrates and glycoproteins.

## INTRODUCTION

Dental plaque is a multispecies bacterial biofilm that forms on the surface of teeth and is one of the primary factors causing periodontitis (1, 2). *Fusobacterium nucleatum* (subspecies *nucleatum*) is a Gram-negative fusiform anaerobe and a major commensal bacterium in the human oral cavity that is predominantly found in subgingival dental plaque (3, 4). *F. nucleatum* also contributes to the progression of periodontitis as a periodontal pathogen. For example, this bacterium adheres to and invades gingival epithelial cells via a “zipping” mechanism (5), a process mediated by surface proteins, such as Fap2 and RadD, leading to the production of inflammatory cytokines (6). In addition to its pathogenic role, *F. nucleatum* is well known as a bridging organism in dental plaque. It adheres to several Gram-positive early colonizers on tooth surfaces, such as *Streptococcus* and *Actinomyces* species (7, 8), and subsequently to late colonizers, primarily Gram-negative anaerobes, including major periodontal pathogens, such as *Porphyromonas gingivalis*, *Tannerella forsythia*, and *Treponema denticola* (9–12). Thus, *F. nucleatum* contributes to dental plaque development through its interspecies interactions (coaggregation) (4, 13). Since the progression of periodontitis is strongly associated with the localization of late colonizers, bacterial coaggregation mediated by *F. nucleatum* plays a crucial role in oral health (14).

A major periodontal pathogen, *P. gingivalis* (15) shows enhanced growth under oxygenated and CO_2_-depleted conditions when cocultured with *F. nucleatum* (16). Furthermore, *F. nucleatum* facilitates the bacterial invasion of *P. gingivalis* into gingival epithelial and endothelial cells (17). In mouse models, co-infection with *F. nucleatum* and other periodontal pathogens results in significantly more severe bone loss than infection with a single species (18, 19). The interspecies coaggregation interactions of *F. nucleatum* promote biofilm formation. *F. nucleatum* is capable of forming biofilm on collagen-coated plates, and coculture with *P. gingivalis* significantly promotes biofilm formation compared to monocultures (20). Similarly, coculture of *F. nucleatum* with *T. forsythia* synergistically promotes biofilm formation via the *T. forsythia* (21). *F. nucleatum* establishes mutualistic relationships with *Veillonella* species and *Aggregatibacter actinomycetemcomitans*, enabling efficient mixed-species biofilm formation in saliva (22).

*F. nucleatum* is also recognized as a pathogen associated with systemic diseases, including premature/stillbirth and colorectal/breast cancers (23–26). Through transient bacteremia in patients with periodontal disease, *F. nucleatum* can infect vascular endothelial cells and facilitate bacterial invasion into blood vessels by disrupting intercellular adherence via interactions between its surface-exposed autotransporter protein FadA and E-cadherin expressed on endothelial cells, leading to systemic dissemination (27, 28). Furthermore, *F. nucleatum* can bind to overexpressed _D_-galactose-β (1–3)-*N*-acetyl-_D_-galactosamine (Gal-GalNAc) on cancer cells using the galactose-binding protein Fap2, facilitating colonization of carcinomatous lesions (23–25). Additionally, Fap2 suppresses T-cell and NK-cell activities through binding to the TIGIT receptor (23, 29), thereby promoting cancer progression (24, 25).

Several studies have reported that the surface proteins of *F. nucleatum* function as coaggregation factors with various bacterial species. *F. nucleatum* possesses eight high-molecular-weight autotransporter proteins (over 200 kDa) on its outer membrane (7). Among these autotransporters, RadD has been identified as an arginine-inhibitable coaggregation factor with *Streptococcus sanguinis*, *Streptococcus gordonii*, *Streptococcus oralis*, and *Actinomyces naeslundii* (7, 8). Additionally, RadD of *F. nucleatum* subspecies *polymorphum* has affinity for SpaP (also named Pa*c*, P1, or Antigen I/II), a cell wall-anchored protein of the cariogenic pathogen *Streptococcus mutans* (30). Fap2 and CmpA were identified as coaggregation factors for *P. gingivalis* and *S. gordonii*, respectively (8, 10). In addition to autotransporters, the porin-like protein FomA was also identified as a coaggregation factor with *P. gingivalis* (14).

*A. actinomycetemcomitans* is a Gram-negative facultative anaerobe associated with periodontitis, particularly aggressive periodontitis (31). It possesses several virulence factors, such as lipopolysaccharide (LPS) (32), pilus (33), leukotoxin (32, 34), and cytolethal distending toxin (32, 35). *A. actinomycetemcomitans* is classified into seven serotypes (a, b, c, d, e, f, and g) on the basis of the antigenicity of O-polysaccharide (O-PS) regions in LPS (36–39). Serotypes a, b, and c are frequently isolated from periodontitis cases (40, 41). Notably, serotype b JP2 clone strains are associated with aggressive periodontitis due to leukotoxin overproduction (42). Serotype a exhibits relatively low pathogenicity in periodontitis, whereas serotype c is predominantly found in chronic periodontitis (40). Serotypes d, e, and f are less frequently isolated from periodontitis patients (40, 41). Serotype g represents a newly isolated strain from chronic periodontitis (38). *A. actinomycetemcomitans* is also associated with systemic diseases, including endocarditis (43), Alzheimer’s disease (44), and brain abscess (45).

Previous studies have investigated the coaggregation properties of *A. actinomycetemcomitans* with *F. nucleatum*. Kolenbrander et al. demonstrated that *A. actinomycetemcomitans* serotype b strains coaggregate with *F. nucleatum* strains (46). Rupani et al. reported that the *A. actinomycetemcomitans* serotype f strain CU1060N coaggregates with *F. nucleatum* PK1594 and that this coaggregation is inhibited by the addition of galactose (47). Furthermore, Karched et al. reported that the *A. actinomycetemcomitans* serotype d strain SA269 coaggregates with the *F. nucleatum* subspecies *polymorphum* ATCC 10953 (48).

In the present study, we characterized the coaggregation activity of *F. nucleatum* and different serotypes of *A. actinomycetemcomitans*. We found that two autotransporter proteins, Fap2 and CmpA, expressed on the outer membrane of *F. nucleatum* mediated coaggregation with *A. actinomycetemcomitans* serotypes b and d, respectively.

## RESULTS

### *F. nucleatum* coaggregates with *A. actinomycetemcomitans* strains of serotypes b and d

**Fig 1:**
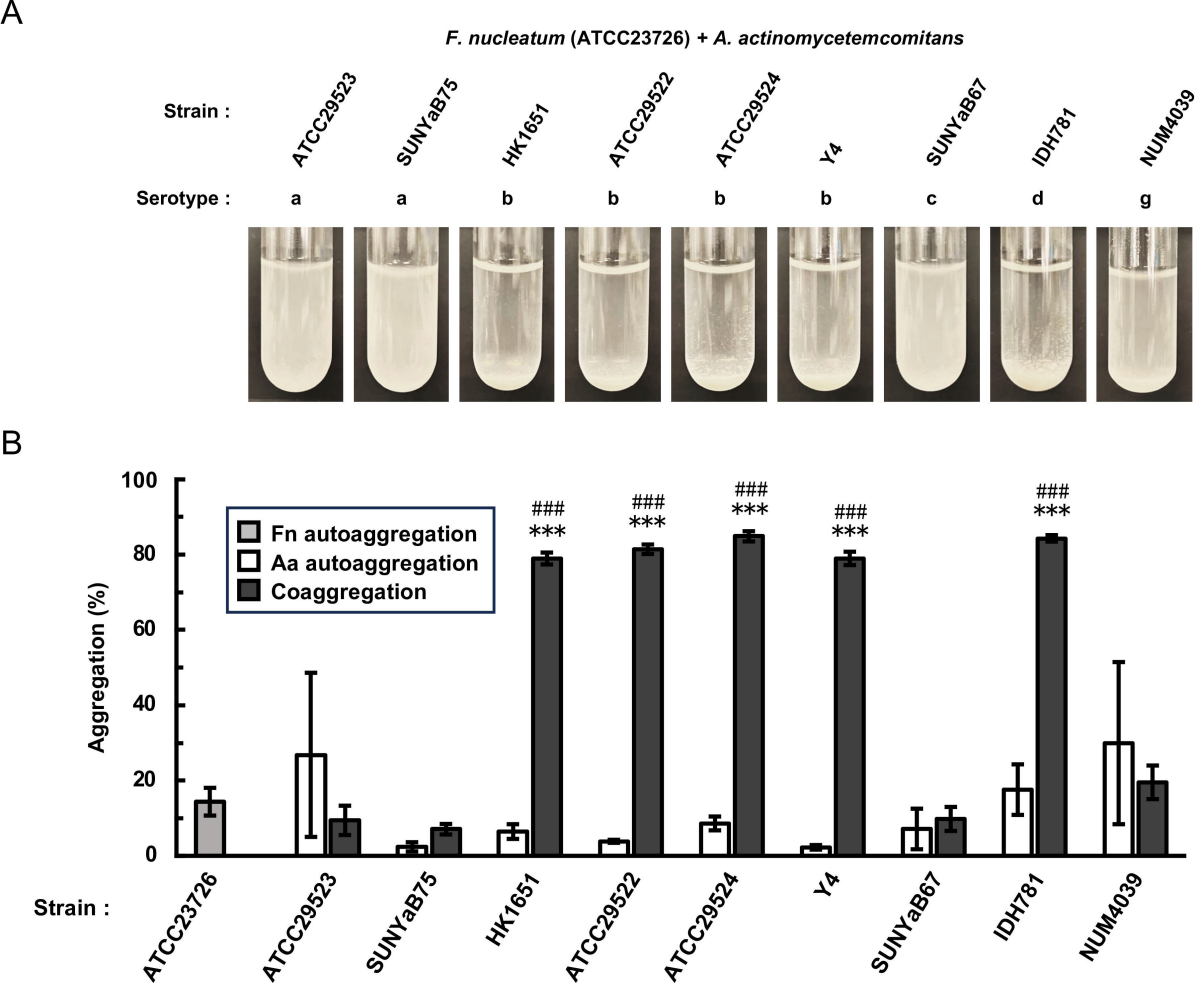
Coaggregation between *F. nucleatum* ATCC 23726 and *A. actinomycetemcomitans* strains. *F. nucleatum* ATCC23726 and *A. actinomycetemcomitans* strains were cultured until the stationary phase and resuspended in coaggregation buffer to an OD_660_ of 2.0. (**A**) Suspensions of *F. nucleatum* and *A. actinomycetemcomitans* were mixed in equal volumes and incubated at 37℃ for 150 min. Representative images are shown. (**B**) Suspensions of *F. nucleatum* and *A. actinomycetemcomitans* were mixed in equal volumes and incubated at 37℃ for 10 min. After gentle centrifugation to precipitate the aggregated cells, the turbidity of the supernatant was measured at OD_660_. The degree of aggregation was quantified by the percentage reduction in the OD_660_ compared with the initial OD_660_. Data are presented as the means ± SDs of five independent experiments. Significant changes in aggregation were determined using Tukey’s test. *** indicates a significant difference between the autoaggregation of *F. nucleatum* and coaggregation (*P* < 0.001). ### indicates a significant difference between the autoaggregation of *A. actinomycetemcomitans* and coaggregation (*P* < 0.001). Fn, *F. nucleatum*; Aa, *A. actinomycetemcomitans*.

The coaggregation properties were examined between two *F. nucleatum* strains (ATCC23726 and ATCC25586) (7, 49) and nine *A. actinomycetemcomitans* strains representing serotypes a, b, c, d, and g (Table 1). Both *F. nucleatum* strains strongly aggregated at the bottom of the test tubes when mixed with *A. actinomycetemcomitans* serotype b strains (HK1651, ATCC29522, ATCC29524, and Y4) and a serotype d strain (IDH781) (Fig. 1A; Fig. S1A). Although ATCC29523 (serotype a) and NUM4039 (serotype g) also aggregated in test tubes (Fig. 1A; Fig. S1A), these *A. actinomycetemcomitans* strains demonstrated single-species aggregation (autoaggregation) (Fig. S2). The results of a quantitative aggregation assay revealed 14% autoaggregation of *F. nucleatum* ATCC23726 and 2% to 29% autoaggregation of *A. actinomycetemcomitans* strains (Fig. 1B). In the coaggregation assay, the aggregation percentage was approximately 80% when either strain of *F. nucleatum* was mixed with *A. actinomycetemcomitans* serotype b or d (Fig. 1B; Fig. S1B). These coaggregation percentages were significantly greater than their respective autoaggregation percentages (Fig. 1B and Fig. S1B). Among serotypes a, b, c, d, and g, we concluded that *F. nucleatum* specifically coaggregated with *A. actinomycetemcomitans* serotypes b and d.

**TABLE 1:** *A. actinomycetemcomitans* strains used in this study

Strain	Serotype	Reference
ATCC29523	a	(50)
SUNYaB75	a	(51)
HK1651	b	(52)
ATCC29522	b	(53)
ATCC29524	b	(54)
Y4	b	(55)
SUNYaB67	c	(54)
IDH781	d	(56)
NUM4039	g	(57)

### *F. nucleatum* coaggregates with *A. actinomycetemcomitans* serotypes b and d by binding to O-antigen polysaccharides

**Fig 2:**
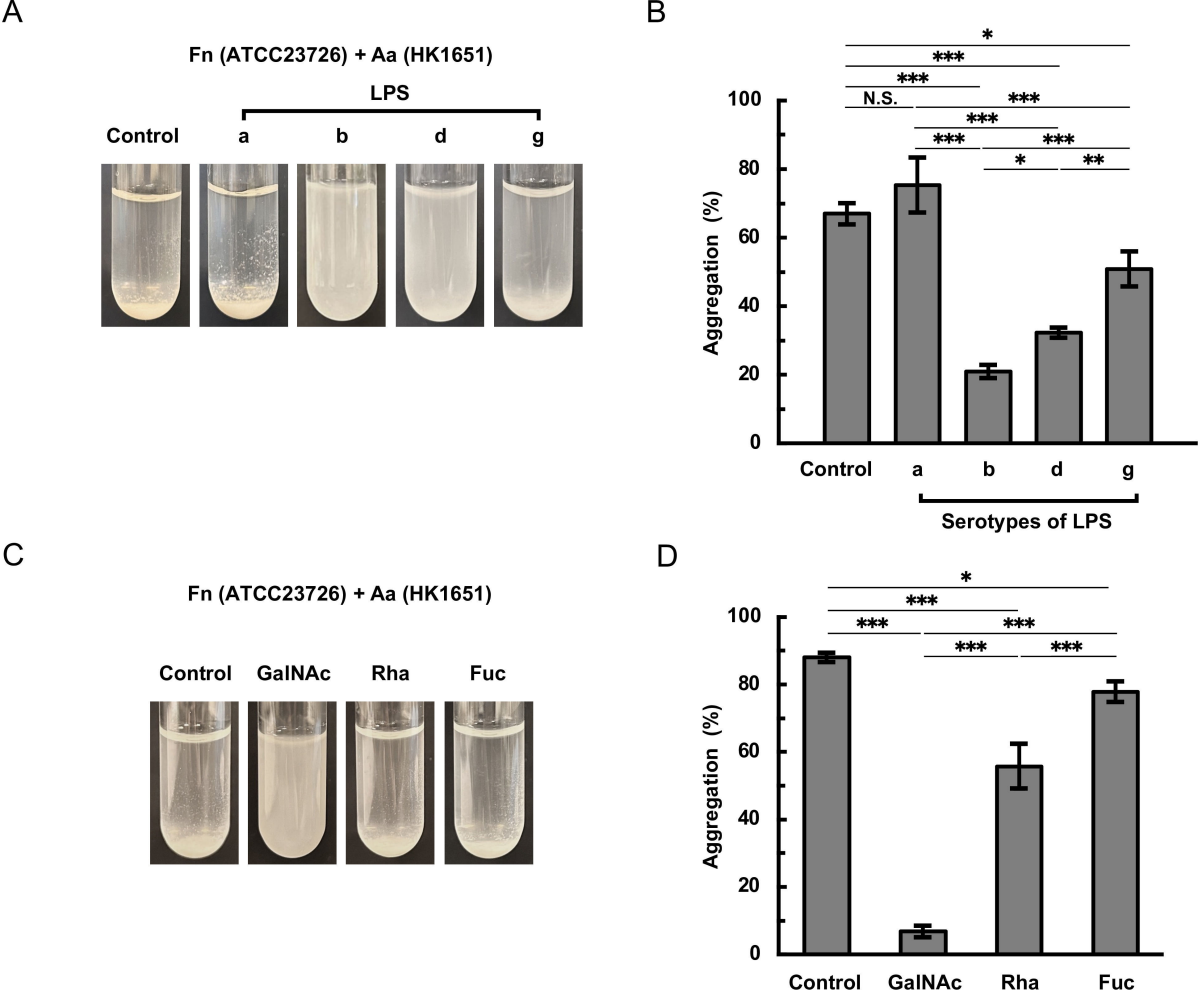
Inhibitory effects of LPS fractions and sugars on coaggregation between *F. nucleatum* and *A. actinomycetemcomitans* serotype b strain. *F. nucleatum* ATCC23726 and *A. actinomycetemcomitans* HK1651 (serotype b) were cultured until the stationary phase, resuspended in coaggregation buffer containing either *A. actinomycetemcomitans* LPS (0.5  mg/mL) or 5 mM sugar, and adjusted to an OD_660_ of 2.0. Equal volumes of each bacterial suspension were mixed and incubated at 37℃ for 150  min. Representative images are shown in panels **A** (LPS) and **C** (sugar). For quantitative analysis, the same mixtures were incubated at 37℃ for 10 min and centrifuged to precipitate the aggregated cells. The turbidity of the supernatant was measured at OD_660_. The degree of aggregation was quantified by the percentage reduction in the OD_660_ compared with the initial OD_660_. Results from the suspensions in LPS- and sugar-containing buffers are shown in panels **B** and **D**, respectively. Data are presented as the means ± SDs of three independent experiments. Significant differences among samples were determined using Tukey’s test (*, *P* < 0.05; **, *P* < 0.01; ***, *P* < 0.001; N.S., not significant). Control, coaggregation buffer without LPS or sugar; GalNAc, *N*-acetyl-_D_-galactosamine; Rha, _L_-rhamnose; Fuc, _D_-fucose. Fn, *F. nucleatum*; Aa, *A. actinomycetemcomitans*.

**Fig 3:**
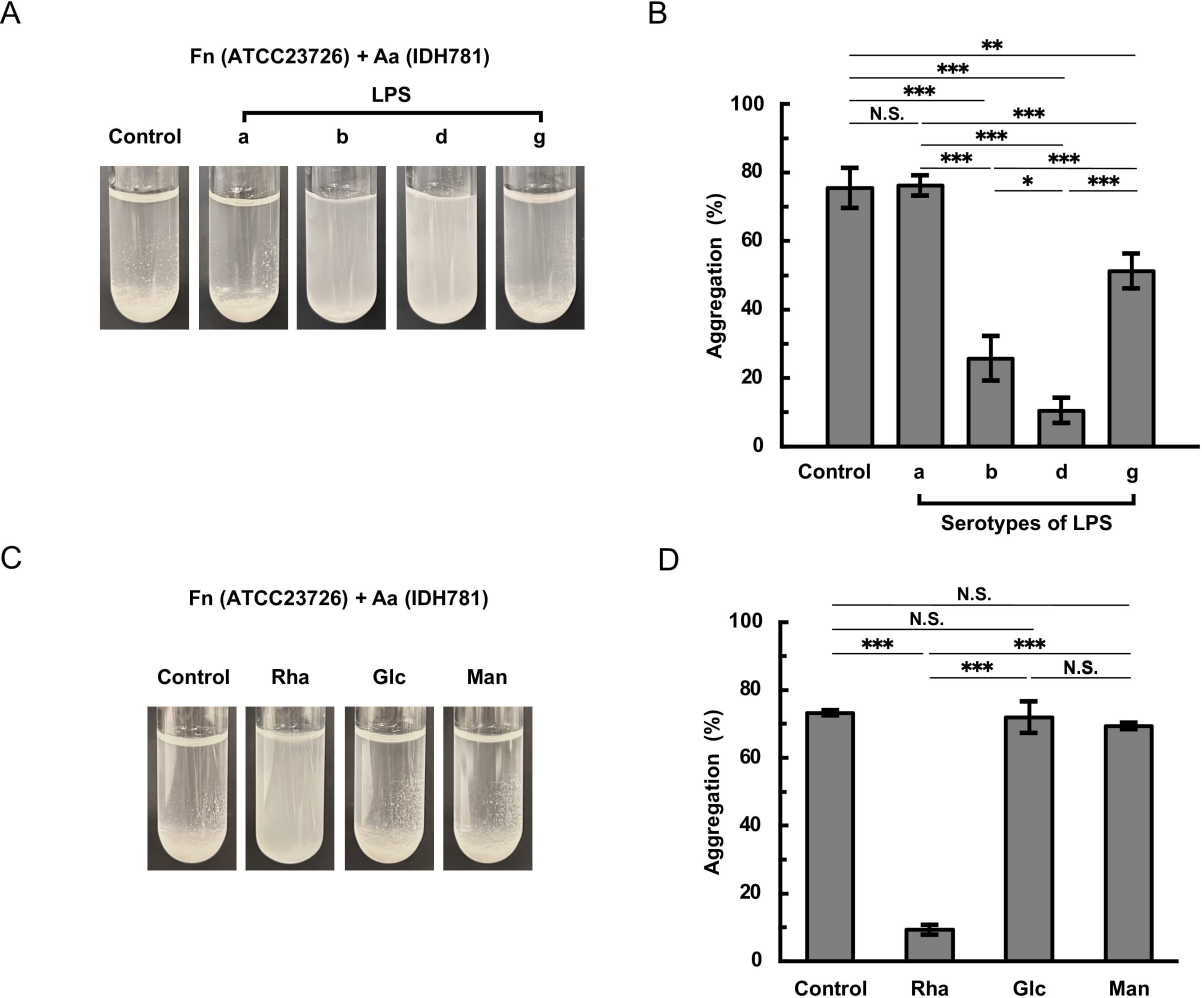
Inhibitory effects of LPS fractions and sugars on coaggregation between *F. nucleatum* and *A. actinomycetemcomitans* serotype d strain. *F. nucleatum* ATCC23726 and *A. actinomycetemcomitans* IDH781 (serotype d) were cultured until the stationary phase, resuspended in coaggregation buffer containing either *A. actinomycetemcomitans* LPS (0.5  mg/ml) or 5 mM sugar, and adjusted to an OD_660_ of 2.0. Equal volumes of each bacterial suspension were mixed and incubated at 37℃ for 150  min. Representative images are shown in panels **A** (LPS) and **C** (sugar). For quantitative analysis, the same mixtures were incubated at 37℃ for 10 min and centrifuged to precipitate the aggregated cells. The turbidity of the supernatant was measured at OD_660_. The degree of aggregation was quantified by the percentage reduction in the OD_660_ compared with the initial OD_660_. Results from the suspensions in LPS- and sugar-containing buffers are shown in panels **B** and **D**, respectively. Data are presented as the means ± SDs of three independent experiments. Significant differences among samples were determined using Tukey’s test (*, *P* < 0.05; **, *P* < 0.01; ***, *P* < 0.001; N.S., not significant). Control, coaggregation buffer without LPS or sugar; Rha, _L_-rhamnose; Glc, _D_-glucose; Man, _D_-mannose. Fn, *F. nucleatum*; Aa, *A. actinomycetemcomitans*.

To identify factors involved in the coaggregation between *F. nucleatum* and *A. actinomycetemcomitans* serotypes b and d strains, we analyzed LPS fractions extracted from *A. actinomycetemcomitans* strains since *A. actinomycetemcomitans* serotypes are classified on the basis of the antigenicity of their O-PS regions. The structures of *A. actinomycetemcomitans* O-PS are shown in Fig. S3. Compared with that of the vehicle control without LPS, the coaggregation between *F. nucleatum* ATCC23726 and *A. actinomycetemcomitans* HK1651 (serotype b) was inhibited by buffer containing LPS from serotype b, d, or g, while negligibly affected by LPS from serotype a (Fig. 2A). LPS from both serotypes b and d showed strong inhibition, while LPS from serotype g showed slight inhibition (Fig. 2A). Quantitative analysis showed that the coaggregation was reduced by 70%, 52%, and 25% in the presence of LPS from serotypes b, d, and g, respectively (Fig. 2B). Since the O-PS of *A. actinomycetemcomitans* serotype b contains _D_-fucose, _L_-rhamnose, and *N*-acetyl-_D_-galactosamine (39) (Fig. S3), we tested the inhibitory effects of these sugars on the coaggregation. The coaggregation of *F. nucleatum* ATCC23726 with *A. actinomycetemcomitans* HK1651 was strongly inhibited by buffer containing *N*-acetyl-_D_-galactosamine (Fig. 2C). Quantitative analysis revealed that the coaggregation was significantly reduced by 93%, 37.5%, and 11% in the presence of *N*-acetyl-_D_-galactosamine, _L_-rhamnose, and _D_-fucose, respectively (Fig. 2D). Similarly, the coaggregation between *F. nucleatum* ATCC23726 and *A. actinomycetemcomitans* IDH781 (serotype d) was also inhibited by buffer containing LPS tested, except for LPS from serotype a. Notably, LPS from both serotypes d and b showed strong inhibition, although the pattern of inhibition was reversed compared to that observed with HK1651 (Fig. 3A). Quantitative analysis showed that the coaggregation was significantly reduced by 67%, 87%, and 32% in the presence of LPS extracted from *A. actinomycetemcomitans* serotypes b, d, and g, respectively (Fig. 3B). The O-PS of *A. actinomycetemcomitans* serotype d consists of _D_-glucose, _D_-mannose, and _L_-rhamnose (36) (Fig. S3). Among the sugars present in serotype d O-PS, _L_-rhamnose significantly inhibited their coaggregation (Fig. 3CD). None of these sugars affected the autoaggregation of either *F. nucleatum* or *A. actinomycetemcomitans* strains (Fig. S4).

### *F. nucleatum* coaggregates with *A. actinomycetemcomitans* serotypes b and d through autotransporter proteins

**Fig 4:**
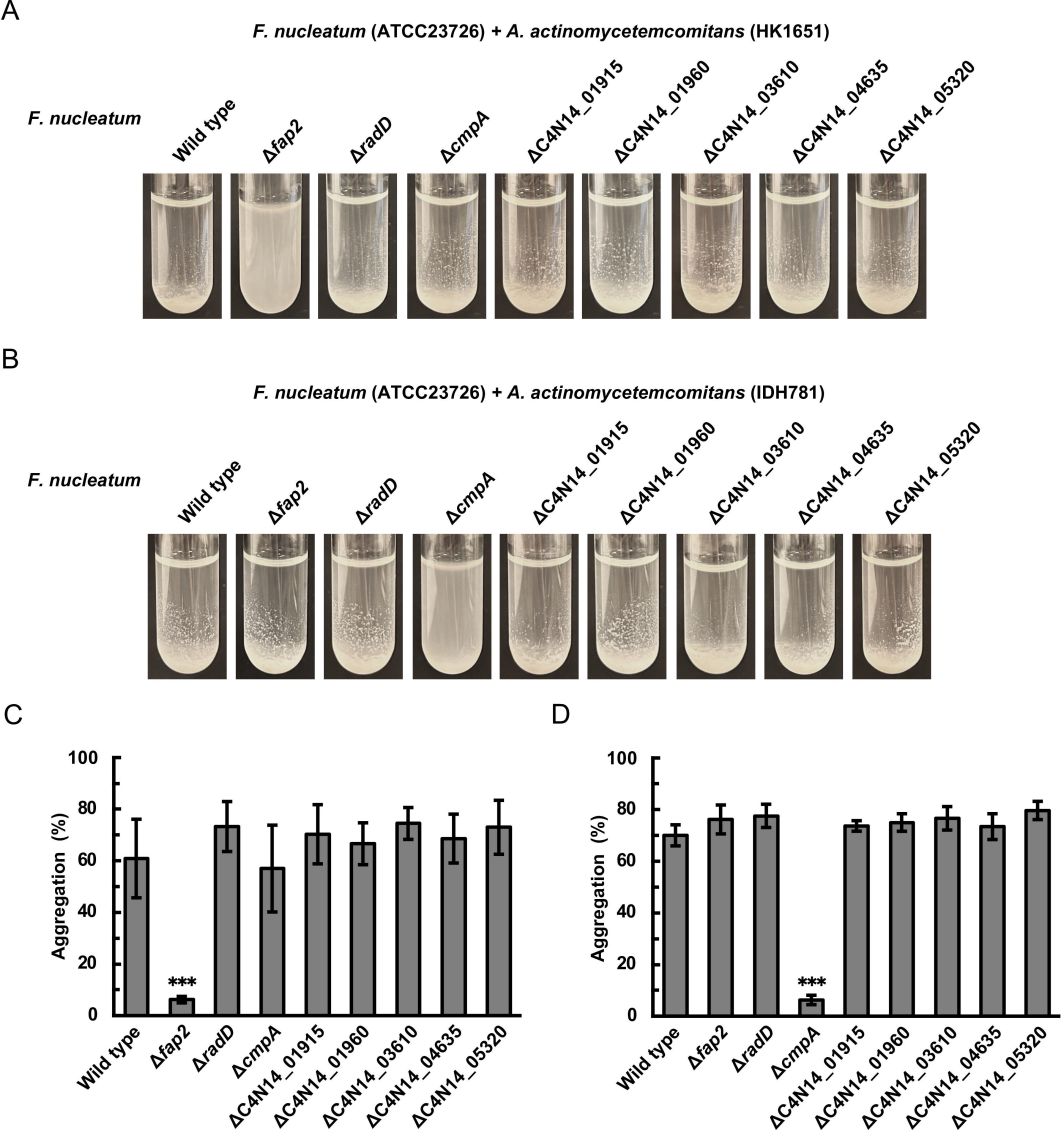
Coaggregation between *F. nucleatum* strains lacking autotransporter proteins and *A. actinomycetemcomitans. F. nucleatum* ATCC23726, its mutants lacking eight distinct autotransporter genes, *A. actinomycetemcomitans* HK1651 (serotype b), and *A. actinomycetemcomitans* IDH781 (serotype d) were cultured until the stationary phase and resuspended in coaggregation buffer to an OD_660_ of 2.0. Suspensions of *F. nucleatum* strains and either HK1651 or IDH781 were mixed in equal volumes and incubated at 37°C for 150  min. Representative images are shown in panels **A** (*F. nucleatum* and HK1651) and **B** (*F. nucleatum* and IDH781). For quantitative analysis, the same mixtures were incubated at 37°C for 10 min and centrifuged to precipitate the aggregated cells. The turbidity of the supernatant was measured at OD_660_. The degree of aggregation was quantified by the percentage reduction in the OD_660_ compared with the initial OD_660_. Results for coaggregation between *F. nucleatum* and HK1651 or IDH781 are shown in panels **C** and **D**, respectively. Data are presented as the means ± SDs of three independent experiments. Significant differences compared with the wild type were determined using Dunnett’s test (***, *P* < 0.001).

Since autotransporter proteins in *F. nucleatum* are known to mediate coaggregation with oral bacterial species (7, 8), we constructed mutant strains for eight distinct autotransporter genes. The autotransporter genes were disrupted by the insertion of a plasmid, which is a derivative of pJIR750, using single crossover homologous recombination (Fig. S5A). To confirm the gene disruption, PCR was performed using primers designed to amplify the junctional regions between the inserted plasmid and chromosomal DNA (Fig. S5B). The wild-type ATCC23726 and mutants were analyzed for their ability to coaggregate with *A. actinomycetemcomitans* serotypes b and d (Fig. 4A and B). Compared with that of the wild type, the aggregation percentage of the *fap2* mutant and *A. actinomycetemcomitans* HK1651 (serotype b) was significantly reduced by 90% (Fig. 4C). The *fap2* mutant also exhibited significantly reduced coaggregation with other *A. actinomycetemcomitans* serotype b strains (ATCC29522, ATCC29524, and Y4) (Fig. S6). Similarly, compared with that of the wild type, the aggregation percentage of the *cmpA* mutant and *A. actinomycetemcomitans* IDH781 (serotype d) was significantly reduced by 91% (Fig. 4D).

### Coaggregation between *F. nucleatum* and *A. actinomycetemcomitans* serotype b or d promotes biofilm formation

**Fig 5:**
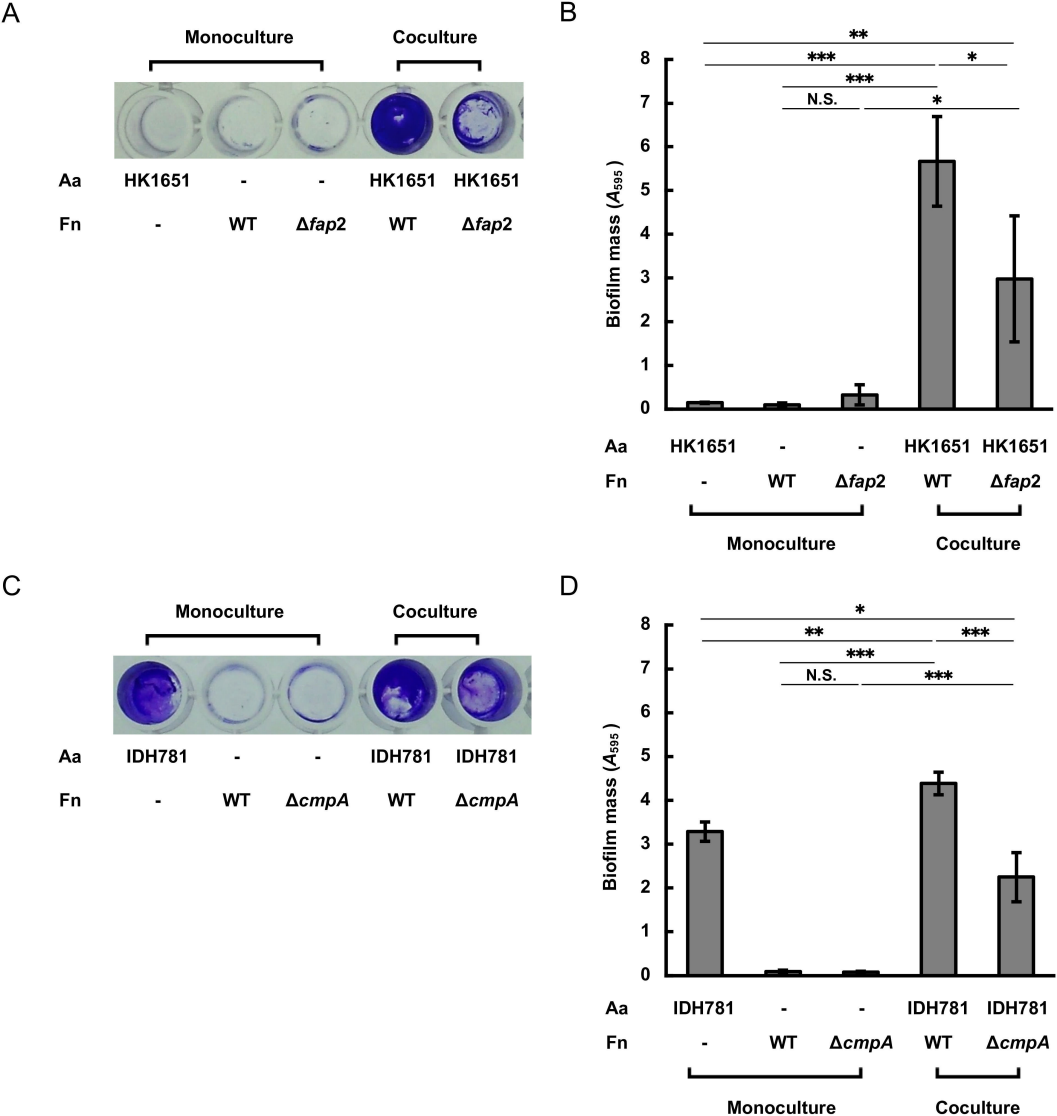
Biofilm formation in cocultures of *F. nucleatum* strains with *A. actinomycetemcomitans* serotype b or d. *F. nucleatum* ATCC23726 and its *fap2*- and *cmpA*-mutant strains, as well as *A. actinomycetemcomitans* HK1651 (serotype b) and IDH781 (serotype d), were cultured until the stationary phase. Equal volumes of *F. nucleatum* and *A. actinomycetemcomitans* suspensions (OD_660_ = 1.0) were mixed in TSPC + AAGM broth in a 96-well flat-bottom plate and incubated anaerobically at 37℃ for 24 h. After incubation, planktonic cells were removed, and biofilms were stained with crystal violet. Representative images of monoculture biofilms and coculture biofilms of *F. nucleatum* with HK1651 and IDH781 are shown in panels **A** and **C**, respectively. Quantification of the biofilm mass is shown in panels **B** (*F. nucleatum* with HK1651) and **D** (*F. nucleatum* with IDH781). Data are presented as the means ± SDs of three independent experiments. Significant differences were determined using Tukey’s test. Symbols indicate statistically significant comparisons between biologically relevant sample pairs (*, *P* < 0.05; **, *P* < 0.01; ***, *P* < 0.001; N.S., not significant). Fn, *F. nucleatum*; Aa, *A. actinomycetemcomitans*.

To analyze the synergistic effect of the coaggregation on bacterial growth, cocultures of *F. nucleatum* ATCC23726 and *A. actinomycetemcomitans* HK1651 (serotype b) or IDH781 (serotype d) were examined. In liquid culture, the growth of ATCC23726 cocultured with HK1651 or IDH781 was not increased compared to that of the corresponding *fap2* or *cmpA* mutant cocultures, respectively (Fig. S7). When cultured individually, HK1651, ATCC23726, and its isogenic *fap2* mutant did not form detectable biofilms on polystyrene plates after 24 h of monoculture under our experimental conditions. In contrast, robust biofilm formation was observed in the coculture of ATCC23726 and HK1651 (Fig. 5A). When the *fap2* mutant was cocultured with HK1651, the biofilm mass was significantly reduced by 47% compared to that observed in the coculture of ATCC23726 and HK1651 (Fig. 5B). In comparison, IDH781 formed biofilms even in monoculture (Fig. 5C), and the coculture with ATCC23726 resulted in a 33% increase in biofilm mass compared to the IDH781 monoculture (Fig. 5D). When the *cmpA* mutant was cocultured with IDH781, the biofilm mass was significantly reduced by 49% compared to that observed in the coculture of ATCC23726 and IDH781 (Fig. 5D).

## DISCUSSION

In the present study, we demonstrated that coaggregation between *F. nucleatum* and *A. actinomycetemcomitans* serotype b (HK1651) was strongly inhibited by the addition of an LPS fraction purified from *A. actinomycetemcomitans* serotype b (Fig. 2A and B). Furthermore, *F. nucleatum* strains lacking the autotransporter protein Fap2 did not coaggregate with *A. actinomycetemcomitans* serotype b (Fig. 4A and C; Fig. S6), suggesting that their coaggregation occurs through binding between LPS and Fap2. Among the sugars composing the O-PS region of LPS, *N*-acetyl-_D_-galactosamine had the strongest inhibitory effect on coaggregation (Fig. 2C and D). This finding is consistent with previous studies by Weiss et al., who demonstrated that coaggregation between the *F. nucleatum* strain PK1594 and the *A. actinomycetemcomitans* serotype b strain (JP2) was a galactose-sensitive reaction (58). Additionally, Rupani et al. observed that the *A. actinomycetemcomitans* serotype f strain CU1060N, which contains _L_-rhamnose and *N*-acetyl-_D_-galactosamine in its O-PS (Fig. S3), coaggregated with *F. nucleatum* PK1594, and this coaggregation was inhibited by LPS extracted from CU1060N (47). They also identified a mutant strain of CU1060N that lacked coaggregation activity with *F. nucleatum* PK1594 due to a transposon insertion in the gene cluster responsible for serotype f O-PS synthesis. These findings suggest that the *N*-acetyl-_D_-galactosamine residue of O-PS found in serotypes b and f is crucial for coaggregation with *F. nucleatum*. In this study, we identified Fap2 as a coaggregation factor for *A. actinomycetemcomitans* serotype b (Fig. 4A and C). Fap2, an outer membrane autotransporter protein, is known to bind to tumor-expressed Gal-GalNAc (23) and has been identified as a galactose-sensitive coaggregation factor with *Enterococcus faecalis* (59). Therefore, the coaggregation between *A. actinomycetemcomitans* serotype b and *F. nucleatum* likely occurs through the interaction between Fap2 and the *N*-acetyl-_D_-galactosamine residue in the O-PS region of LPS.

We also demonstrated that coaggregation between *F. nucleatum* and the *A. actinomycetemcomitans* serotype d strain (IDH781) was inhibited by the addition of the LPS fraction extracted from *A. actinomycetemcomitans* serotype d (Fig. 3A and B). Among the sugars comprising the O-PS of serotype d LPS, _L_-rhamnose significantly inhibited their coaggregation (Fig. 3C and D). Moreover, the *F. nucleatum* strain lacking *cmpA* did not coaggregate with *A. actinomycetemcomitans* serotype d (Fig. 4B and D). Although CmpA has been reported to mediate coaggregation with several *Streptococcus* species and *A. naeslundii* in an arginine-inhibitable manner (7, 8), the addition of 5 mM _L_-arginine to the coaggregation reaction between *F. nucleatum* ATCC23726 and *A. actinomycetemcomitans* IDH781 had slight inhibitory effects (9.5%) (Fig. S8). These results suggest that coaggregation between *F. nucleatum* and *A. actinomycetemcomitans* serotype d occurs predominantly through an arginine-independent mechanism. Notably, *S. gordonii*, which coaggregates with *F. nucleatum* via CmpA, also contains rhamnose residues in the heptasaccharide repeat of its coaggregation receptor polysaccharide (60, 61). These findings suggest that the coaggregation between *F. nucleatum* and *A. actinomycetemcomitans* serotype d is mediated through the interaction between CmpA and the rhamnose residue of LPS.

Our results revealed that coaggregation between *F. nucleatum* and *A. actinomycetemcomitans* strains was suppressed by the addition of LPS fractions purified from *A. actinomycetemcomitans* serotypes b, d, and g (Fig. 2AB and 3AB). Structural analysis of these serotypes revealed distinct O-PS compositions (Fig. S3): Serotype b O-PS consists of a disaccharide (_D_-fucose and _L_-rhamnose) backbone with an *N*-acetyl-_D_-galactosamine side-chain residue on the _L_-rhamnose residue (39); serotype d O-PS consists of a trisaccharide (_D_-glucose, _D_-mannose, _D_-mannose) backbone with an _L_-rhamnose side-chain on the middle _D_-mannose residue (36); and serotype g O-PS consists of a disaccharide (_D_-glucose, _L_-rhamnose) without side-chain residues (38). Given that CmpA functions as a rhamnose-inhibitable coaggregation factor of *F. nucleatum* (Fig. 3C and D and Fig. 4B through D) and that rhamnose residues are common among the O-PSs of serotypes b, d, and g (Fig. S3), we propose that the inhibitory effects observed with different LPS fractions may result from rhamnose residues blocking CmpA. This hypothesis is supported by the partial inhibition of coaggregation between *F. nucleatum* and *A. actinomycetemcomitans* serotype b by the addition of _L-_rhamnose (Fig. 2D).

Several lines of evidence suggest that the side-chain sugar residues of O-PS play a more significant role in coaggregation than the backbone sugars. (I) The inhibitory effect of serotype g LPS on coaggregation between *F. nucleatum* and *A. actinomycetemcomitans* serotype b was lower than that of serotype d LPS (Fig. 2B). (II) _L-_Rhamnose had weaker inhibitory effects than *N*-acetyl-_D_-galactosamine on coaggregation between *F. nucleatum* and *A. actinomycetemcomitans* serotype b (Fig. 2D). (III) Serotypes b and g LPS had weaker inhibitory effects than did serotype d LPS on coaggregation between *F. nucleatum* and *A. actinomycetemcomitans* serotype d (Fig. 3B). (IV) Despite containing the _L-_rhamnose residue in its backbone sugars, the *A. actinomycetemcomitans* serotype g strain (NUM4039) showed no coaggregation with *F. nucleatum* (Fig. 1). Since sugar residues located on side chains are spatially more exposed and thus are considered to be more accessible to binding sites, autotransporter proteins of *F. nucleatum* may preferentially target these side-chain sugars in the O-PS. The lack of coaggregation with serotype g supports the hypothesis that side-chain sugars are primary determinants of experimentally detectable coaggregation. In serotype b, coaggregation with *F. nucleatum* was primarily mediated by *N*-acetyl-_D_-galactosamine residues located on the O-PS side chains. However, partial inhibition was also observed upon the addition of backbone rhamnose from serotype g (Fig. 2B). Coaggregation between *F. nucleatum* and *A. actinomycetemcomitans* serotype d was also partially inhibited upon the addition of backbone rhamnose from serotype b or g (Fig. 3B). These findings suggest that backbone sugars may also contribute to the interaction with *F. nucleatum*.

Our coculture biofilm formation assay demonstrated that inactivation of *fap2* or *cmpA* in *F. nucleatum* resulted in reduced biofilm formation with *A. actinomycetemcomitans* serotype b (HK1651) or serotype d (IDH781), respectively (Fig. 5). In contrast, monocultures of *F. nucleatum* or HK1651 did not form detectable biofilms on uncoated polystyrene plates under our experimental conditions. Several published studies have demonstrated biofilm formation by *F. nucleatum* on plates pre-coated with agents, such as saliva, gelatin, or collagen (3, 20, 62). However, our experiments were conducted without any coating agents, which likely explains the absence of biofilm formation in *F. nucleatum* monocultures. In cocultures, coaggregated bacterial cells settled at the bottom of the plates more efficiently than non-aggregated cells in monocultures. This likely increased the number of cells in contact with plate surfaces, partly contributing to the robust biofilm formation observed in coculture. Unlike HK1651, IDH781 forms substantial biofilms in monoculture. During coculture, it is possible that the IDH781 biofilm may have served as a scaffold that facilitated mixed-species biofilm development with *F. nucleatum*. These findings provide important insights from a clinical perspective, as mixed-species biofilms formed by *F. nucleatum* and *A. actinomycetemcomitans* developed more rapidly than those formed by either species in monoculture. Therefore, investigating strategies to inhibit this coaggregation may contribute to improving oral hygiene and the prevention of periodontal diseases.

In conclusion, we elucidated the molecular mechanisms underlying coaggregation between two periodontal pathogens, *F. nucleatum* and *A. actinomycetemcomitans* serotype b or d. Given the increasing recognition of *F. nucleatum* and *A. actinomycetemcomitans* in systemic diseases beyond periodontitis, our findings provide valuable insights into a mechanism by which these periodontal pathogens colonize host tissues through specific molecular interactions.

## MATERIALS AND METHODS

### Bacterial strains and culture conditions

*F. nucleatum* strains ATCC23726 and ATCC25586 were cultured in TSPC medium (3% Trypticase soy broth [Becton, Dickinson and Company, Franklin Lakes, NJ, USA] and 1% Bacto peptone [Becton, Dickinson and Company]) and separately autoclaved with 0.25% _L_-cysteine hydrochloride monohydrate (FUJIFILM Wako Pure Chemical Corporation, Osaka, Japan), at 37°C under anaerobic conditions using the GasPack system AnaeroPack KENKI (Mitsubishi Gas Chemical Co., Osaka, Japan). To culture the *F. nucleatum* mutant strains, thiamphenicol (FUJIFILM Wako Pure Chemical Corporation) was added to the TSPC medium at a concentration of 5 μg/mL.

The *A. actinomycetemcomitans* strains used in this study are listed in Table 1. These strains were cultured in *Aggregatibacter actinomycetemcomitans* growth medium (AAGM) (63) containing 2.5% TSB, 0.6% yeast extract (Becton, Dickinson and Company), 1.0% _D_-glucose (FUJIFILM Wako Pure Chemical Corporation), and 0.4% sodium bicarbonate (Nacalai Tesque, Inc., Kyoto, Japan) at 37℃ in ambient air supplemented with 5% CO_2_.

The *Escherichia coli* strain XL10-Gold (Stratagene, San Diego, CA, USA) was cultured in Luria-Bertani (LB) medium (Nacalai Tesque, Inc.) at 37℃ with shaking (140 rpm). Chloramphenicol (5 µg/mL, FUJIFILM Wako Pure Chemical Corporation) was added to the LB medium to maintain the plasmid pJIR750 (64).

### Construction of *F. nucleatum* mutant strains

Gene inactivation in *F. nucleatum* was performed according to a modified version of a previously described protocol (65). An internal DNA fragment (approximately 1,400 bp) of the target gene was amplified by PCR using primers containing SpeI and EcoRI restriction sites (Nippon Gene Co. Ltd., Tokyo, Japan). The amplified DNA fragment was electrophoresed, purified from an agarose gel using the GENECLEAN III Kit (MP Biomedicals, LLC, Irvine, CA, USA), and digested with SpeI and EcoRI. The *Clostridium perfringens* and *E. coli* shuttle vector pJIR750 was also digested with SpeI and EcoRI, electrophoresed, and purified from an agarose gel. The digested fragment and pJIR750 were ligated using T4 DNA ligase (Takara Bio Inc., Kusatsu, Japan) and transformed into chemically competent *E. coli* XL10-Gold cells. The constructed plasmid was validated by PCR using the primers shown in Table S1 and purified using the Quantum prep Plasmid Miniprep Kit (Bio-Rad Laboratories, Inc., San Francisco, CA, USA).

To prepare *F. nucleatum* competent cells, *F. nucleatum* ATCC 23726 was cultured in 8 mL of TSPC at 37°C for 24 h under anaerobic conditions. The culture was then diluted with 42 mL of TSPC and incubated at 37°C until the optical density at 660 nm (OD_660_) reached 0.3. The cells were chilled on ice and centrifuged at 3,000 × *g* for 10 min, after which the pellet was resuspended in 20 mL of ice-cold water. After two additional washes with water, the cells were resuspended in 10% glycerol (FUJIFILM Wako Pure Chemical Corporation) and kept on ice until use.

For transformation, 1 µg of plasmid DNA was mixed with 40 µL of the *F. nucleatum* competent cells and incubated on ice for 10 min. The mixture was electroporated using a Gene Pulser (Bio-Rad Laboratories, Inc.) at 2.5 kV and 25 µF. Following electroporation, the cells were incubated in 10 mL of TSPC at 37°C for 3 h anaerobically, and appropriate dilutions were plated on TSPC agar containing thiamphenicol (5 µg/mL). The plates were incubated at 37°C for five days under anaerobic conditions. Chromosomal DNA was extracted from the obtained colonies, and plasmid insertion into target genes was confirmed by PCR using the primers listed in Table S1.

### Preparation of *A. actinomycetemcomitans* LPS

LPS was purified from the *A. actinomycetemcomitans* strains HK1651, IDH781, NUM4039, and ATCC29523 using a modified phenol‒water method (66, 67). Bacteria were cultured in 500 mL of Trypticase soy broth containing 0.6% yeast extract for 24  h at 37℃ with 5% CO_2_. The cells were harvested by centrifugation at 3,000 × *g* for 20  min, washed three times with 10 mL of phosphate-buffered saline (PBS), and lyophilized using an FDU-1200 (EYELA, Tokyo, Japan). The dried bacterial cells were suspended in distilled water (17.5 times the dry weight), mixed with an equal volume of water-saturated phenol (FUJIFILM Wako Pure Chemical Corporation), and incubated at 65℃ for 30  min with shaking. After centrifugation at 8,000 × *g* for 20  min, the aqueous phase was collected, and the extraction was repeated with fresh distilled water. The combined aqueous phases were dialyzed three times against 5 L of distilled water using a dialysis tube (MWCO: 3,000) (Ieda Boueki, Tokyo, Japan). The dialyzed samples were treated with five units of DNase I RT-grade (Nippon Gene Co. Ltd.) and 5  µg of RNase A (Takara Bio Inc.) in DNase buffer (Nippon Gene Co. Ltd.) at 37℃ for 1  h. Subsequently, 0.5% sodium dodecyl sulfate (FUJIFILM Wako Pure Chemical Corporation) and 25  µg of proteinase K (Nacalai Tesque, Inc.) were added, and the samples were incubated at 56℃ for 1  h, followed by overnight incubation at 37℃ with shaking. The samples were re-extracted with water-saturated phenol and dialyzed as described above. The purified LPS fraction was lyophilized and dissolved in coaggregation buffer (CAB: 1 mM Tris-HCl [pH 7.5], 0.1 mM CaCl_2_, 0.1 mM MgCl_2_, 150 mM NaCl) at a concentration of 0.5  mg/mL. All reagents to make CAB were purchased from FUJIFILM Wako Pure Chemical Corporation.

### Aggregation assay

The *F. nucleatum* and *A. actinomycetemcomitans* strains were cultured in their respective growth media under appropriate conditions. Stationary phase cells (*F. nucleatum*; OD_660_ = 1.0, *A. actinomycetemcomitans*; OD_660_ = 0.85) were harvested by centrifugation (3,000 × *g*, 10 min) and resuspended in CAB. The cell suspensions were adjusted to an OD_660_ of 2.0 in test tubes. For the autoaggregation assays, 2 mL of single bacterial suspension was used; for the coaggregation assays, equal volumes of *F. nucleatum* and *A. actinomycetemcomitans* suspensions were mixed to a total volume of 2 mL. The tubes were incubated at 37℃ for 150 min, and bacterial aggregation was observed as precipitation at the bottom of the test tubes under gravity.

For quantitative analysis, 1 mL of bacterial suspension (OD_660_ = 2.0) was prepared in CAB in 1.5 mL tubes. The initial OD_660_ (0_t_) was measured by transferring a small portion of the suspension to a polystyrene cuvette (12.5 × 12.5 × 45 mm) (AS ONE CORPORATION, Osaka, Japan) using an Ultrospec 2000 UV/visible spectrophotometer (Pharmacia Biotech Ltd., Cambridge, United Kingdom). The remaining suspension was incubated at 37℃ for 10 min in a block incubator. Following gentle centrifugation (100 × *g*, 1 min) to precipitate the aggregated cells, the OD_660_ of the supernatant (10_t_) was measured.

To evaluate the effects of sugars on aggregation and coaggregation, both types of aggregation assays were performed using CAB containing the following sugars at 5 mM: _L_-rhamnose (Nacalai Tesque, Inc.), _D_-mannose (FUJIFILM Wako Pure Chemical Corporation), *N*-acetyl-_D_-galactosamine (Combi-Blocks, Sandiego, CA, USA), _D_-fucose (FUJIFILM Wako Chemical Corporation), or _D_-glucose (FUJIFILM Wako Chemical Corporation).

To analyze the effects of purified *A. actinomycetemcomitans* LPS on coaggregation, both types of aggregation assays were performed using CAB containing purified LPS (0.5  mg/mL). For quantitative analysis of the coaggregation in the presence of LPS, we performed small-scale experiments. A total of 100 µL of mixed bacterial suspensions (*F. nucleatum : A. actinomycetemcomitans* = 1:1, OD_660_ = 2.0) were prepared in CAB containing LPS. The initial OD_660_ (0_t_) was measured using a 96-well round polystyrene plate (Thermo Scientific, Inc., Waltham, USA) and a SpectraMax 340PC384 Microplate Reader (Molecular Devices, LLC, Tokyo, Japan). After 10 min of incubation at 37℃ and gentle centrifugation (100 × *g*, 1 min), the OD_660_ of the supernatant (10_t_) was measured.

The percentage of aggregation was calculated using the following formula: aggregation (%) ＝ 100 × (0_t_ − 10_t_) / 0_t_.

### Coculture biofilm assay

*F. nucleatum* and *A. actinomycetemcomitans* strains were cultured in their respective growth media under appropriate conditions. Stationary-phase cells were harvested by centrifugation (12,000 × *g*, 1 min) and resuspended in TSPC + AAGM (3% TSB, 0.6% yeast extract, 1.0% _D_-glucose, 0.4% sodium bicarbonate, 1% Bacto peptone, and 0.25% _L_-cysteine hydrochloride monohydrate) and adjusted to an OD_660_ of 1.0. To examine biofilm formation, 10 µL of the bacterial suspension (coculture: 5 µL of *F. nucleatum* and 5 µL of *A. actinomycetemcomitans*) was added to 200 µL of TSPC + AAGM in a 96-well flat-bottom polystyrene microplate (AS ONE Corporation) and incubated anaerobically at 37 ˚C for 24 h. After incubation, the culture medium and planktonic cells were removed by aspiration, and the wells were washed three times with 100 µL of PBS by rotation (120 rpm; PlateSpin3, Kubota Corporation, Tokyo, Japan) for 5 min. Biofilms were fixed with methanol for 10 min, stained with 0.1% crystal violet (Merck KGaA, Darmstadt, Germany) for 10 min, and washed three times with 150 µL of water. The bound dye was eluted with 100 µL of ethanol, and biofilm mass was quantified by measuring absorbance at 595 nm (*A*_595_) using a SpectraMax 340PC384 Microplate Reader.

### Statistical analysis

Statistical analyses were performed using EZR (version 1.27) (Saitama Medical Center, Jichi Medical University), a graphical user interface for R (R Foundation for Statistical Computing, Vienna, Austria). Significant differences were determined using one-way analysis of variance (ANOVA) followed by Tukey’s and Dunnett’s multiple-comparison tests or Student’s *t* test. Differences were considered statistically significant at *P* < 0.05.

## Data Availability

This study did not generate any unique data sets or code. All data supporting the findings of this study are available within the article and its supplemental material.

## SUPPLEMENTAL MATERIAL

Supplemental material aem.01132-25-s0001.pdf Table S1; Fig. S1 to S8.

ASM does not own the copyrights to Supplemental Material that may be linked to, or accessed through, an article. The authors have granted ASM a non-exclusive, world-wide license to publish the Supplemental Material files. Please contact the corresponding author directly for reuse.
