# Supplementary material for: The outer membrane autotransporters Fap2 and CmpA facilitate specific coaggregation between Fusobacterium nucleatum and Aggregatibacter actinomycetemcomitans serotypes b and d
Source: Appl Environ Microbiol. 2025 Oct 20;91(11):e01132-25. doi: 10.1128/aem.01132-25 (PMC12628838; doi:10.1128/aem.01132-25)
Supplement: Supplemental material — Table S1; Fig. S1 to S8. [file aem.01132-25-s0001.pdf]

The outer membrane autotransporters Fap2 and CmpA facilitate specific coaggregation between *Fusobacterium nucleatum* and *Aggregatibacter actinomycetemcomitans* serotypes b and d

Yumika Tanaka[a,b], Yuichi Oogai[a#], Airi Matsumoto[a], Kazuyuki Noguchi[b], Masanobu Nakata[a#]

[a]Department of Oral Microbiology, Kagoshima University Graduate School of Medical and Dental Sciences, Kagoshima, Japan
[b]Department of Periodontology, Kagoshima University Graduate School of Medical and Dental Sciences, Kagoshima, Japan

Supplemental Materials

Supplemental Methods

PCR validation of *F. nucleatum* mutant strains

Chromosomal DNA was extracted from candidate mutant strains using the DNeasy Blood & Tissue Kit (QIAGEN, Hilden, Germany). Plasmid insertion into target genes was confirmed by PCR using *Ex Taq* DNA polymerase (Takara Bio Inc., Kusatsu, Japan) and the primers listed in Supplemental Table 1. PCR products were separated by electrophoresis on a 1% agarose (FUJIFILM Wako Pure Chemical Corporation, Osaka, Japan) gel using Mupid-2plus (Takara Bio Inc.) in Tris-borate-EDTA buffer, and stained with ethidium bromide (Nacalai Tesque, Inc., Kyoto, Japan). Gel images were captured using a ChemiDoc MP imaging system (Bio-Rad Laboratories, Inc., San Francisco, CA, USA).

Coculture assay

F. nucleatum and *A. actinomycetemcomitans* strains were cultured in their respective growth media under appropriate conditions. Stationary phase cells were harvested by centrifugation (12,000 × *g*, 1 min) and resuspended in TSPC+AAGM [3% Trypticase soy broth (Becton, Dickinson and Company, Franklin Lakes, NJ, USA), 0.6% yeast extract (Becton, Dickinson and Company), 1.0% D-glucose (FUJIFILM Wako Pure Chemical Corporation, Osaka, Japan), 0.4% sodium bicarbonate (Nacalai Tesque, Inc., Kyoto, Japan), 1% Bacto peptone (Becton, Dickinson and Company), and 0.25% L-cysteine hydrochloride monohydrate (FUJIFILM Wako Pure Chemical Corporation)] and adjusted to an OD_{660} of 1.0. A total of 200 µL of bacterial suspension (coculture: 100 µL of *F. nucleatum* and 100 µL of *A. actinomycetemcomitans*) was added to 5 ml of TSPC+AAGM in a test tube and incubated anaerobically at 37°C. OD_{660} was measured at 4, 6, 8, 10, 12, and 24 h using a spectrophotometer (mini photo 518R, TAITEC Corporation, Saitama, Japan). Tubes were mixed using a vortex mixer for 5 sec prior to each measurement.

Supplemental Table 1. Primers used in this study

Primer	Sequence (5' - 3')
For construction of *F. nucleatum* mutant strains [1]	
Fap2KO-F	AATATTGGAATTCAACCACC
Fap2KO-R	TTCCAACTAAACTAGTTGCTGT
RadDKO-F	GATGCAGAATTCCTATCAGG
RadDKO-R	ACTGCTACTAGTCCTGTTGC
CmpAKO-F	ATTTAAAGAATTCAGTGGGAAG
CmpAKO-R	TCCACTAGTTCCATATAAATTTGCTGTTACT
C4N14_01915KO-F	ATAGAATTCTCAGGAGAAAAAGTTACAGG
C4N14_01915KO-R	TCCACTAGTGTCTACATTTCCAGCATAGA
C4N14_01960KO-F	ACAAGAAAGTATAGGAATTCAT
C4N14_01960KO-R	AACACTAGTATTTCCAGCTCCTAAATTAG
C4N14_03610KO-F	GAACATTGAATTCAAGTGGA
C4N14_03610KO-R	TTCCACTAGTTACTGTTGTAATTGTTCCTTC
C4N14_04635KO-F	TTGGGAGGAATTCTTACTCT
C4N14_04635KO-R	ATAACTAGTACCTGATGTATCATTTCCAT
C4N14_05320KO-F	CTATGAATTCAAGTAAAAATGC
C4N14_05320KO-R	CCTACTAGTTTTATAACTGCATTTGTTGCT
For validation of *F. nucleatum* mutant strains [2]	
Fap2KO-check-R	GTCATTCCTCATTATAATCTCC
RadDKO-check-R	TACATCTGCTCTTGATGTTTC
CmpAKO-check-R	GGTCAGTATAATAGGGTAGAAC
C4N14_01915KO-check-R	TCCATAAGTCTCCTCCTTC
C4N14_01960KO-check-R	CATACTGACCCTCTTCTG
C4N14_03610KO-check-R	TCCTCCTGCTACAAATAAAC
C4N14_04635KO-check-R	GTTGCTGTATTTGTTAATGTAAC
C4N14_05320KO-check-R	ATGAGTGCCAAACATGAC
M13puc rev	AGCGGATAACAATTTCACACAG

[1] The restriction sites are underlined.

[2] The site-specific primers and M13puc rev were used to validate an insertion of pJIR750 into target genes.

Supplemental Fig.1

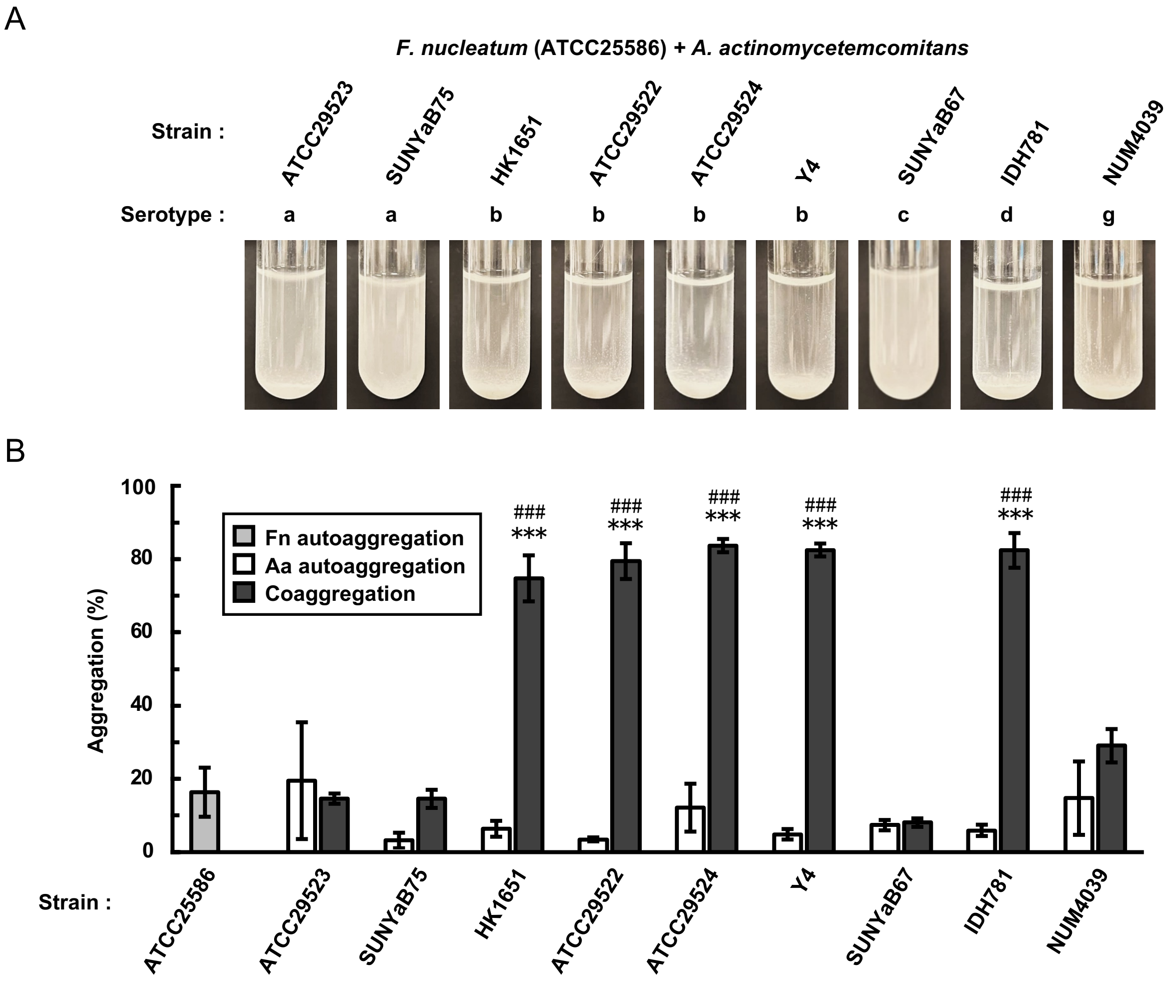

Supplemental Fig. 1. Coaggregation between *F. nucleatum* ATCC25586 and *A. actinomycetemcomitans* strains.

F. nucleatum ATCC25586 and *A. actinomycetemcomitans* strains were cultured until the stationary phase and resuspended in coaggregation buffer to an OD_{660} of 2.0. The suspensions of *F. nucleatum* and *A. actinomycetemcomitans* were mixed in equal volumes and incubated at 37°C for 150 min. (A) Representative images are shown. (B) Suspensions of *F. nucleatum* and *A. actinomycetemcomitans* were mixed in equal volumes and incubated at 37°C for 10 min. After gentle centrifugation to precipitate the aggregated cells, the turbidity of the supernatant was measured at OD_{660}. The degree of aggregation was quantified by the percentage reduction in the OD_{660} compared with the initial OD_{660}. Data are presented as the means ± SDs of five independent experiments. Significant changes in aggregation were determined using Tukey's test. *** indicates a significant difference between the autoaggregation of *F. nucleatum* and coaggregation ($p < 0.001$). ### indicates a significant difference between the autoaggregation of *A. actinomycetemcomitans* and coaggregation ($p < 0.001$). Fn, *F. nucleatum*; Aa, *A. actinomycetemcomitans*.

Supplemental Fig.2

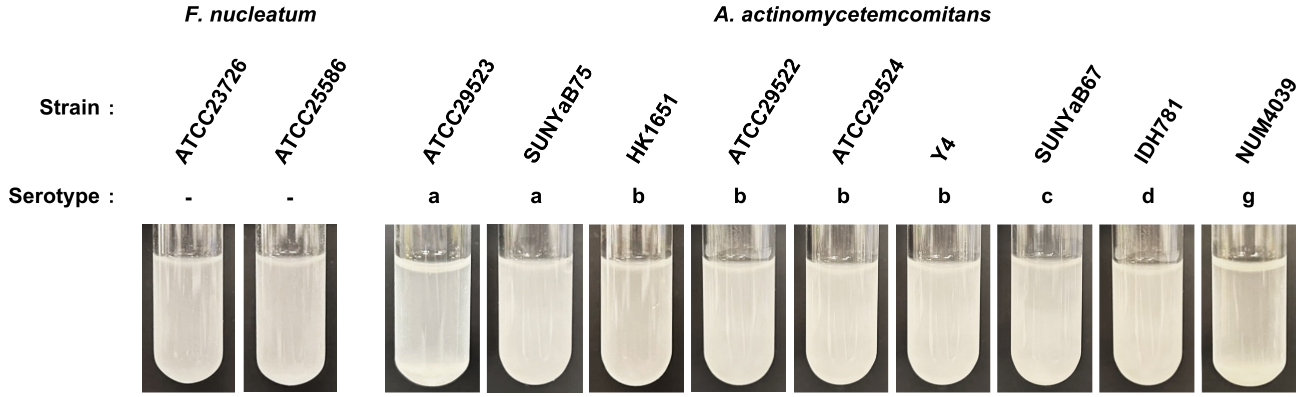

Supplemental Fig. 2. Autoaggregation of *F. nucleatum* and *A. actinomycetemcomitans* strains.

F. nucleatum and *A. actinomycetemcomitans* strains were cultured until the stationary phase and resuspended in coaggregation buffer to an OD_{660} of 2.0. The suspensions were incubated at 37°C for 150 min. Representative images are shown.

Supplemental Fig.3

Serotype a

→3)-*α*-D-6dTal*p*-(1→2)-*α*-D-6dTal*p*-(1→
2
↑
Ac

Serotype b

→3)-*α*-D-Fuc*p*-(1→2)-*α*-L-Rha*p*-(1→
3
↑
1
***β*-D-Gal*p*NAc**

Serotype c

→3)-*α*-L-6dTal*p*-(1→2)-*α*-L-6dTal*p*-(1→
4
↑
Ac

Serotype d

→3)-*β*-D-Glc*p*-(1→4)-*β*-D-Man*p*-(1→4)-α-D-Man*p*-(1→
3
↑
1
α-L-Rha*p*

Serotype e

→4)-*α*-D-Glc*p*NAc-(1→3)-*α*-L-Rha*p*-(1→

Serotype f

→2)-*α*-L-Rha*p*-(1→3)-*α*-L-Rha*p*-(1→
2
↑
1
***β*-D-Gal*p*NAc**

Serotype g

→4)-*α*-D-Glc*p*-(1→3)-*α*-L-Rha*p*-(1→

Supplemental Fig. 3. Structures of *A. actinomycetemcomitans* O-antigen polysaccharides (O-PS).

The O-PS structures of *A. actinomycetemcomitans* serotype a to g were illustrated based on published reports (36-39). D-6dTal*p*, 6-deoxy-D-talopyranose; D-Fuc*p*, D-fucopyranose; L-Rha*p*, L-rhamnopyranose; D-Gal*p*NAc, *N*-acetyl-D-galactopyranose; L-6dTal*p*, 6-deoxy-L-talopyranose; D-Glc*p*, D-glucopyranose; D-Man*p*, D-mannopyranose; D-Glc*p*NAc, *N*-acetyl-D-glucopyranose Ac, Acetyl group.

Supplemental Fig.4

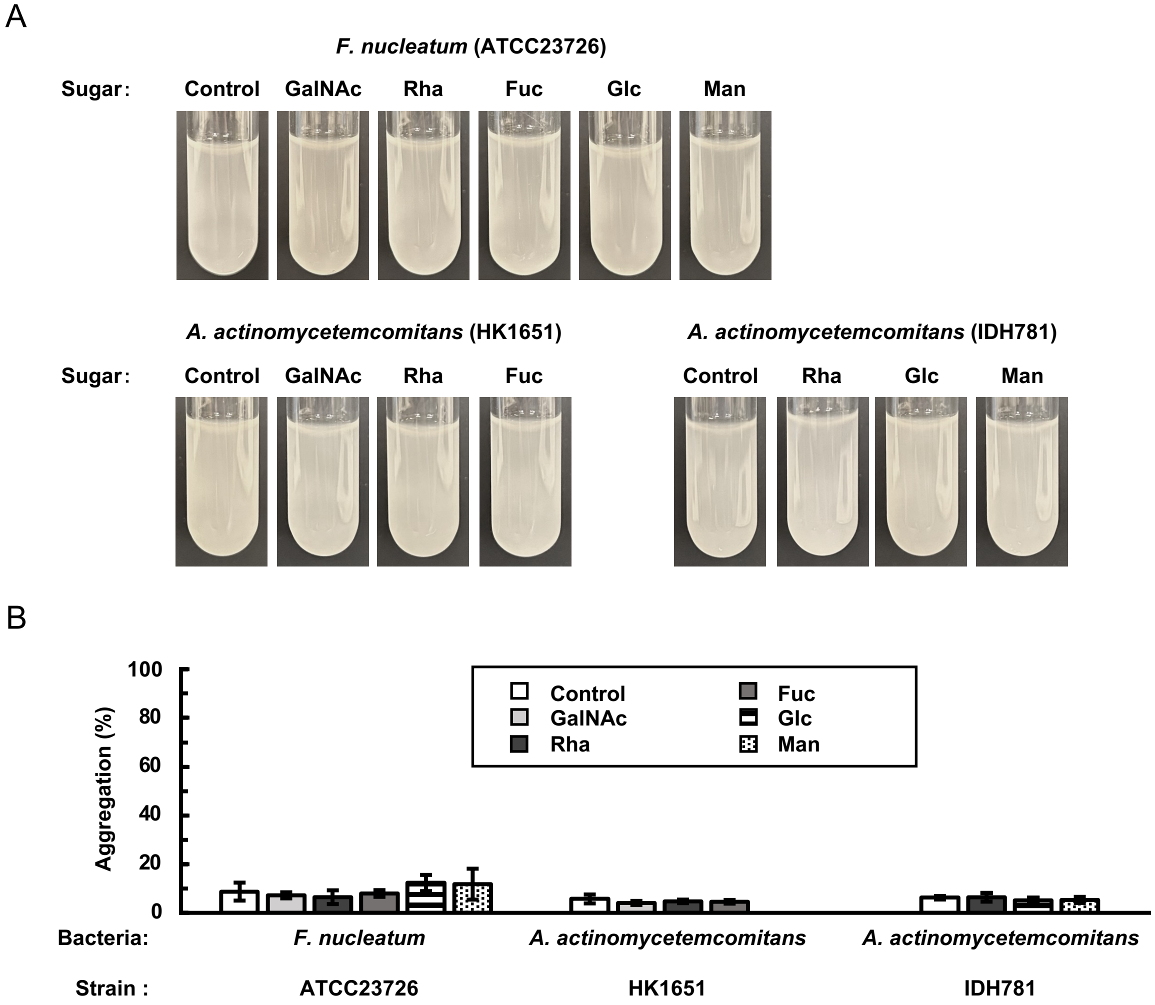

Supplemental Fig. 4. Autoaggregation of *F. nucleatum* and *A. actinomycetemcomitans* strains treated with sugar.

F. nucleatum and *A. actinomycetemcomitans* strains were cultured until the stationary phase, resuspended in coaggregation buffer containing 5 mM sugar, and adjusted to an OD_{660} of 2.0. (A) The suspensions incubated at 37°C for 150 min. Representative images are shown. (B) For quantitative analysis, the same suspensions were incubated at 37°C for 10 min and centrifuged to precipitate the aggregated cells. The turbidity of the supernatant was measured at OD_{660}. The degree of aggregation was quantified by the percentage reduction in the OD_{660} compared with the initial OD_{660}. Data are presented as the means ± SDs of three independent experiments. Statistical significance was not detected among tested strains using Tukey's test. Control, coaggregation buffer without sugar; GalNAc, *N*-acetyl-$_D$-galactosamine; Rha, $_L$-rhamnose; Fuc, $_D$-fucose; Glc, $_D$-glucose; Man, $_D$-mannose.

Supplemental Fig.5

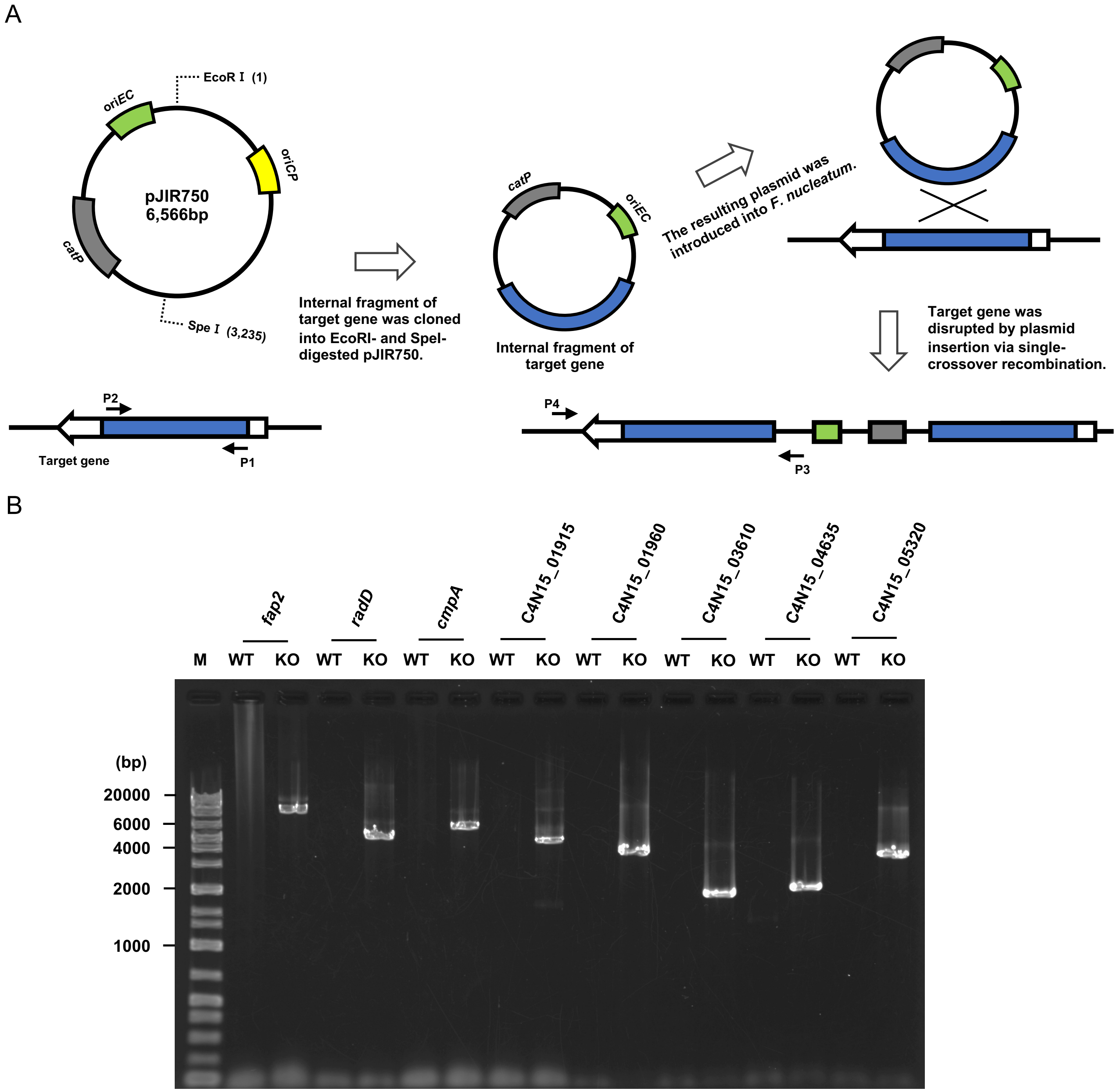

Supplemental Fig. 5. Construction and validation of *F. nucleatum* mutant strains.

(A) Schematic representation of mutant construction by single-crossover plasmid insertion. Details are described in the Materials and Methods section. Black arrows indicate the locations of primers. Nucleotide sequences of the primers are shown in Supplemental Table 1. Primers P1 and P2 were used to amplify an internal DNA fragment of the target gene, while P3 (M13puc rev) and P4 were used to validate of plasmid insertion into the target gene. *catP* encodes chloramphenicol acetyltransferase derived from *Clostridium perfringens.* ori*EC* is the origin of replication used in *Escherichia coli* hosts, and ori*CP* is the origin of replication used in *C. perfringens* hosts. (B) Validation of the mutant construction. PCR products generated from genomic DNA purified from wild type (WT) and each mutant strain (KO) using primers P3 and P4 were separated on a 1% agarose gel and visualized by ethidium bromide staining. M, size marker.

Supplemental Fig.6

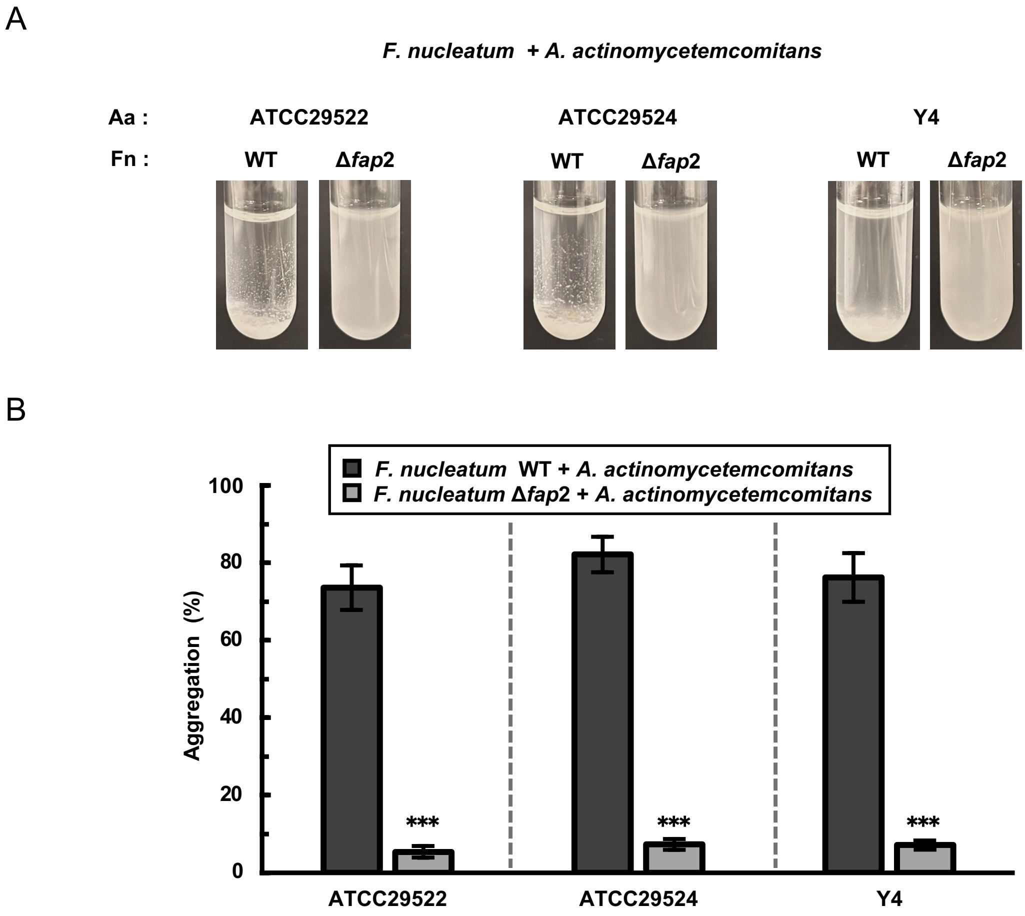

Supplemental Fig. 6. Coaggregation between *F. nucleatum* wild type/*fap*2-mutant strains and *A. actinomycetemcomitans* serotype b strains.

F. nucleatum ATCC23726, its *fap*2 mutant, and three strains of *A. actinomycetemcomitans* serotype b (ATCC29522, ATCC29524, and Y4) were cultured until the stationary phase and resuspended in coaggregation buffer to an OD_{660} of 2.0. (A) Equal volumes of each bacterial suspension were mixed and incubated at 37°C for 150 min. Representative images are shown. (B) For quantitative analysis, the same mixtures were incubated at 37°C for 10 min and centrifuged to precipitate the aggregated cells. The turbidity of the supernatant was measured at OD_{660}. The degree of aggregation was quantified by the percentage reduction in the OD_{660} compared with the initial OD_{660}. Data are presented as the means ± SDs of three independent experiments. Significant differences compared with the wild type were determined using Student's *t* test (***, $p < 0.001$). Fn, *F. nucleatum*; Aa, *A. actinomycetemcomitans*.

Supplemental Fig.7

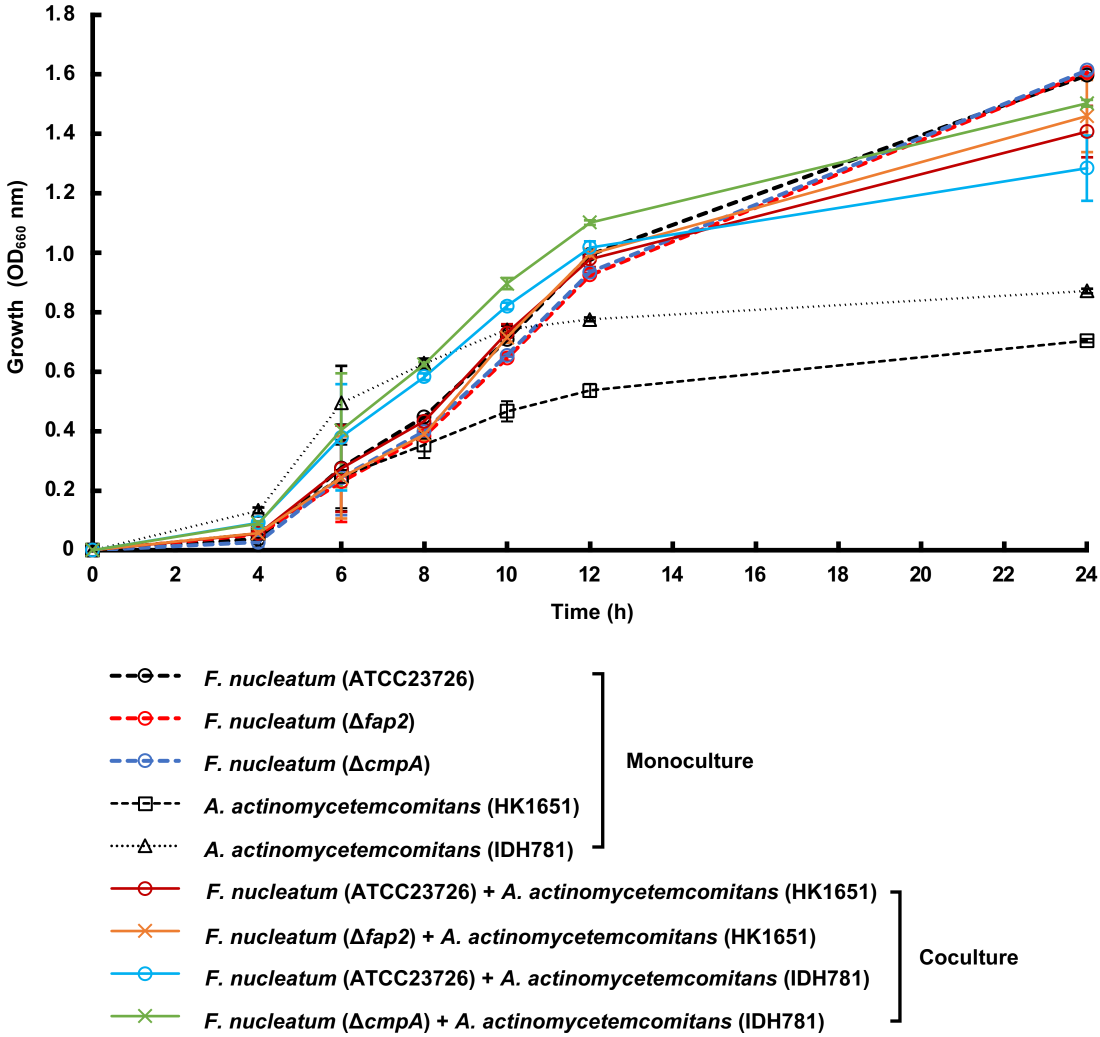

Supplemental Fig. 7. Growth kinetics of cocultures between *F. nucleatum* wild type/*fap2*- or *cmpA*-mutant strains and *A. actinomycetemcomitans* serotype b or d.

F. nucleatum ATCC23726, its *fap2*- and *cmpA*-mutant strain, *A. actinomycetemcomitans* HK1651 (serotype b), and IDH781 (serotype d) were cultured until the stationary phase. Equal volume of *F. nucleatum* and *A. actinomycetemcomitans* cells were cultured in TSPC+AAGM broth and incubated in test tubes at 37°C. Monocultures of each strain were also prepared under the same conditions. The OD_{660} was recorded over 24 h. Data represent the means ± SDs of three independent experiments.

Supplemental Fig.8

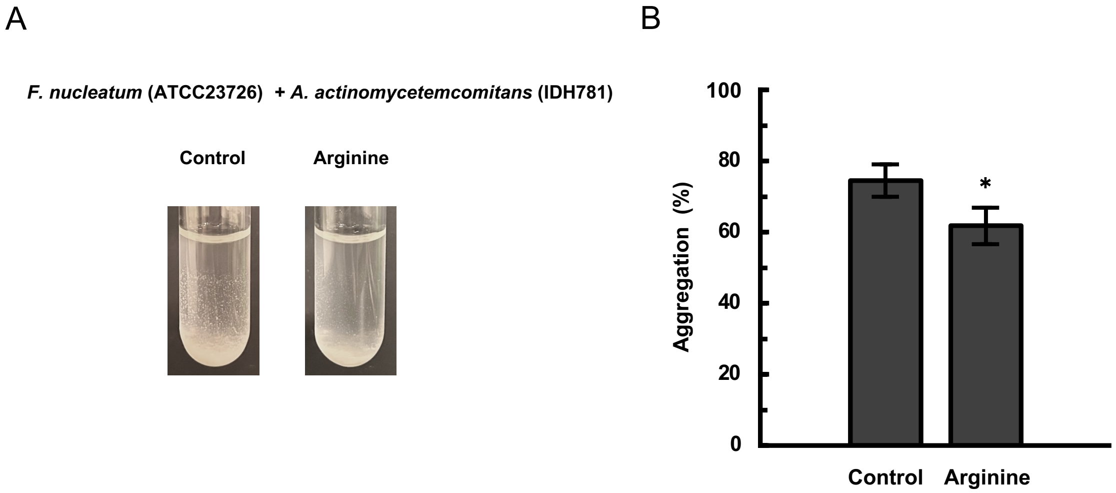

Supplemental Fig. 8 Inhibitory effects of arginine on coaggregation between *F. nucleatum* and *A. actinomycetemcomitans* serotype d.

F. nucleatum ATCC23726 and *A. actinomycetemcomitans* IDH781 (serotype d) were cultured until the stationary phase. These cells were resuspended in coaggregation buffer containing 5 mM L-arginine (FUJIFILM Wako Pure Chemical Corporation) to an OD_{660} of 2.0. (A) Equal volumes of each bacterial suspension were mixed and incubated at 37°C for 150 min. Representative images are shown. (B) For quantitative analysis, the same suspensions were incubated at 37°C for 10 min and centrifuged to precipitate the aggregated cells. The turbidity of the supernatant was measured at OD_{660}. The degree of aggregation was quantified by the percentage reduction in the OD_{660} compared with the initial OD_{660}. Data are presented as the means ± SDs of three independent experiments. Significant differences compared with the control were determined using Student's *t* test (*, $p < 0.05$). Control, coaggregation buffer without arginine.
